# Retraction: Co-Depletion of Cathepsin B and uPAR Induces G0/G1 Arrest in Glioma via FOXO3a Mediated p27^Kip1^ Upregulation

**DOI:** 10.1371/journal.pone.0228690

**Published:** 2020-01-29

**Authors:** The PLOS ONE Editors

After this article [1] was published, the following concerns were raised:

- Figure 1A
  - SNB19, Cathepsin B: Lanes 2 and 3 appear similar.
  - SNB19, uPAR: Lanes 2 and 4 appear similar.
  - U251, Cathespin B: There appears to be a vertical discontinuity after lane 2, and lanes 1 and 3 appear similar.
  - U251, uPAR: There appear to be vertical discontinuities after lanes 2 and 4.
- Figure 2A
  - U251, p-p27 (ser10): There appears to be a sharp horizontal discontinuity at the top of bands in lanes 3–5.
  - U251, p-p27 (Thr187): The background resolution (texture) appears different in lanes 3 and 4 than in lanes 1, 2, 5.
- Figure 2D
  - SNB19, p27: Lanes 4 and 5 appear similar and there are similarities in some background regions in lanes 1 and 2.
  - U251, p27: Lanes 4 and 5 appear similar.
  - U251, GAPDH: Lanes 3 and 4 appear similar to lanes 5 and 6, respectively.
- Figure 3A
  - SNB19, Rb: There appears to be a vertical discontinuity after lane 2.
  - SNB19, GAPDH: There appear to be similarities between upper portions of lanes 1, 2, 4, and between lanes 3, 5.
  - U251, p-CDK2: Lanes 1 and 2 appear similar.
  - U251, CyclinE: There appear to be similarities between image elements in lanes 1 and 2.
  - U251, CyclinD1: The background pattern (texture) appears different between lanes 1–2 and lanes 3–5, and there appears to be a vertical discontinuity after lane 3.
  - U251, CyclinD2: There appear to be similarities between image elements in lanes 4 and 5, and the background pattern (texture) for the lower portions of lanes 1, 2 appears different than that for the remainder of the panel.
  - U251, p-Rb (Ser780): Lanes 1 and 2 appear similar, when horizontally flipped.
  - U251, p21: Lanes 1 and 2 appear similar, and there appear to be dark gray markings below the band in lane 1.
  - U251, Ki67: Lanes 1 and 2 appear similar, when horizontally flipped, except for an area at the top of the band in lane 1.
- Figure 3B
  - U251, IP-CDK2/IB-CyclinE: There appear to be similarities between the upper halves of lanes 1 and 2.
  - U251, αVβ3 (native gel): There appears to be a vertical discontinuity before lane 5.
- Figure 4A
  - SNB19, p-FOXO3a(Ser253): There appear to be similarities between background in lanes 1 and 2.
  - SNB19, p-FOXO3a(Ser318): There appear to be similarities between background in lanes 1 and 2.
  - SNB19, PI3K: There appear to be similarities between background and parts of the bands in lanes 2 and 5.
  - SNB19, PI3K lanes 1, 4 and SNB19, p-Akt lane 2 (flipped horizontally, aspect ratio adjusted) appear similar.
  - There appear to be similarities between SNB19, PI3K lane 3 and SNB19, p-Akt lane 3 (flipped horizontally).
  - SNB19, GAPDH: There appear to be horizontal discontinuities in background above and below the bands, and there appears to be a vertical discontinuity after lane 2.
  - U251, FOXO-3a: When levels are adjusted, there appear to be horizontal and vertical discontinuities around the band in lane 4.
  - U251, p-FOXO3a(Ser253): When levels are adjusted there appears to be a vertical discontinuity after lane 2.
  - U251, p-Akt: There appears to be a vertical discontinuity before lane 3.
- In Figure 6A, the U251 Control/Mock and SV panels appear to show overlapping data.
- In Figure 6B, the SNB19 Control/Mock, Cathepsin B panel (upper region) and SNB19 SV, Ki67 panel (lower region) appear similar.
- Figure S3A
  - FOXO1, left panel: There appear to be similarities between lanes 1, 2, 5, and between lanes 3 and 4.
  - p-FOXO1 (Thr24), left panel: There appear to be similarities between lanes 1 and 3, and between lanes 2 and 4.
  - p-FOXO1 (Ser256), left panel: Areas of lanes 1 and 2 appear similar.
  - p-FOXO1 (Ser319)/FOXO4(Ser262): There appear to be similarities between lanes 1 and 2 of each panel.
  - p-FOXO4 (Ser262): There appear to be similarities between lanes 1, 2, 3 and 5 of the left panel, and when levels are adjusted there appear to be vertical discontinuities after lanes 1, 2, and 4 of the left panel and after lane 1 of the right panel.
  - GAPDH, left panel: There appear to be similarities between lanes 1 and 2.

The above concerns call into question the integrity and reliability of the published results, and so the *PLOS ONE* Editors retract this article.

We attempted to contact the article’s authors to discuss these issues, request the underlying data, and notify them of the retraction decision, but the authors either did not reply or could not be reached.
